# A normal pattern of mitral inflow predicts a better prognosis following cardiovascular events in early advanced-age patients

**DOI:** 10.1038/s41598-022-13802-0

**Published:** 2022-06-10

**Authors:** Tomoyuki Watanabe, Masumi Iwai-Takano, Hiromi Saitoh, Kohko Kanazawa, Takashi Igarashi, Tsuyoshi Fujimiya, Tetsuya Ohira

**Affiliations:** 1 Division of Cardiology and Internal Medicine, Health Co-op Watari Hospital, Fukushima, Japan; 2 Department of Epidemiology, Fukushima Medical University, 1 Hikarigaoka, Fukushima, 960-1295 Japan; 3 Division of Cardiovascular Surgery, Fukushima Medical University, Fukushima, Japan; 4 Fukushima Prefectural General Hygiene Institute, Fukushima, Japan; 5 Clinical Laboratory, Health Co-op Watari Hospital, Fukushima, Japan; 6 Division of Cardiology, Ohta Nishinouchi Hospital, Koriyama, Japan

**Keywords:** Cardiology, Outcomes research

## Abstract

Although a mitral inflow pattern usually changes from a normal pattern to an abnormal relaxation pattern as part of the aging process in healthy people, some early advanced-age individuals maintain a normal pattern. We investigated whether a normal pattern of mitral inflow predicts a better prognosis following cardiovascular (CV) events in early advanced-age patients. We enrolled 425 patients aged 60–65 years with 0.6 < E/A < 1.5. Patients were divided according to their mitral inflow pattern, i.e., a normal pattern group (E/A ≥ 1.0, n = 77) and an abnormal relaxation pattern group (E/A < 1, n = 348), and were evaluated the relationship with CV events. Multivariate regression analysis found that the normal inflow pattern was associated with odds ratios of 0.859 for body mass index (BMI; 95% confidence interval [CI]: 0.778–0.937), 0.529 for hypertension (0.303–0.924), and 0.325 for heart rate (0.228–0.463). During the follow-up period (4.9 ± 1.8 years), the adjusted-hazard ratio was significantly lower in the normal pattern group (HR: 0.119, 95% CI 0.016–0.910). Kaplan–Meier curves showed a higher event-free rate for the normal pattern group than for the abnormal relaxation pattern group (*p* = 0.0292). Normal inflow pattern in early advanced-age patients predicts a better prognosis following CV events.

## Introduction

The hallmark of cardiac aging is a decline of left ventricular diastolic function^1^. Recently, many indices have been proposed to evaluate left ventricular diastolic function, including transmitral inflow pattern, mitral annular velocity by tissue Doppler imaging, and pulmonary vein flow assessment^2,3^. Transmitral inflow is an established tool for the assessment of diastolic function with superior feasibility and reproducible results^4^.

With aging, there is a decline in the peak early mitral inflow velocity (E) and a compensatory increase in atrial inflow velocity (A). Overall, the E/A ratio decreases with age^5^. The mitral inflow pattern changes from a normal pattern (E/A ≥ 1) to an abnormal relaxation pattern (E/A < 1) in healthy subjects without heart disease. The value of E/A is 1 in healthy subjects around the age of 60. The E/A in middle-aged healthy Japanese individuals (50–59 years) is 0.98 ± 0.24 in men and 1.04 ± 0.25 in women^6^.

**Figure 1:**
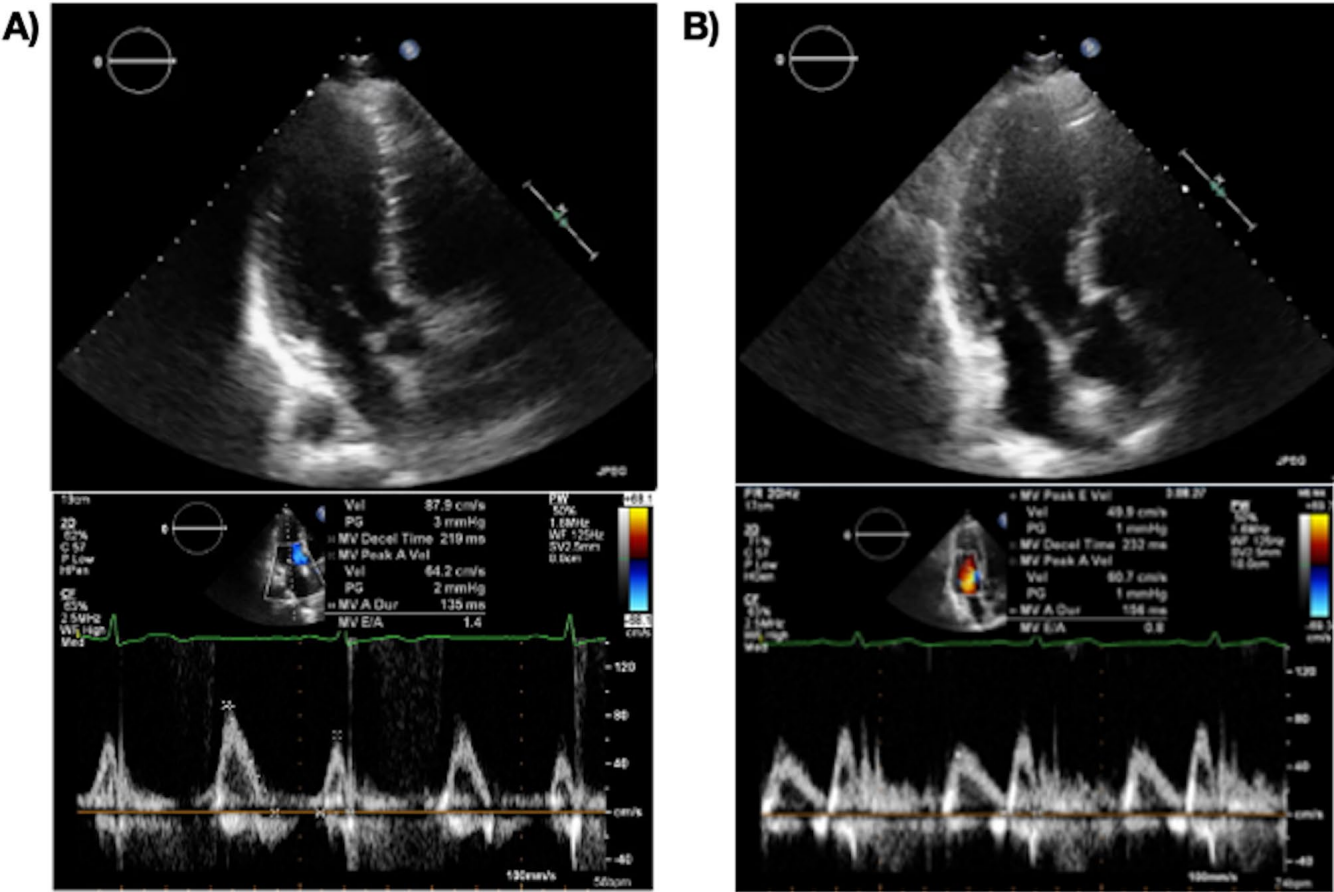
Representative early advanced-age cases with (**A**) normal cardiac size and normal inflow pattern, and, (**B**) abnormal relaxation pattern. Upper panel: Three-camber view. Lower panel: Transmitral inflow by pulse Doppler method.

However, there is considerable variation in the value of E/A even in healthy people. Some in their 50 s have an E/A < 1, while some in their 60 s have an E/A > 1. Some older adults maintain a normal inflow pattern. Figure 1 shows two representative healthy subjects, both aged 63 years with normal heart sizes. While the mitral inflow of one of these subjects has a normal pattern, i.e., the pattern of a “young heart” (Fig. 1A), that of the other has an abnormal relaxation pattern, i.e., an “age-appropriate” pattern (Fig. 1B). It is unknown whether the “young heart” pattern is indicative of prognosis following cardiovascular (CV) events. Therefore, in this study, we investigated whether a normal pattern of mitral inflow predicts a better prognosis following CV events in early advanced-age subjects.

## Methods

### Study population

**Figure 2:**
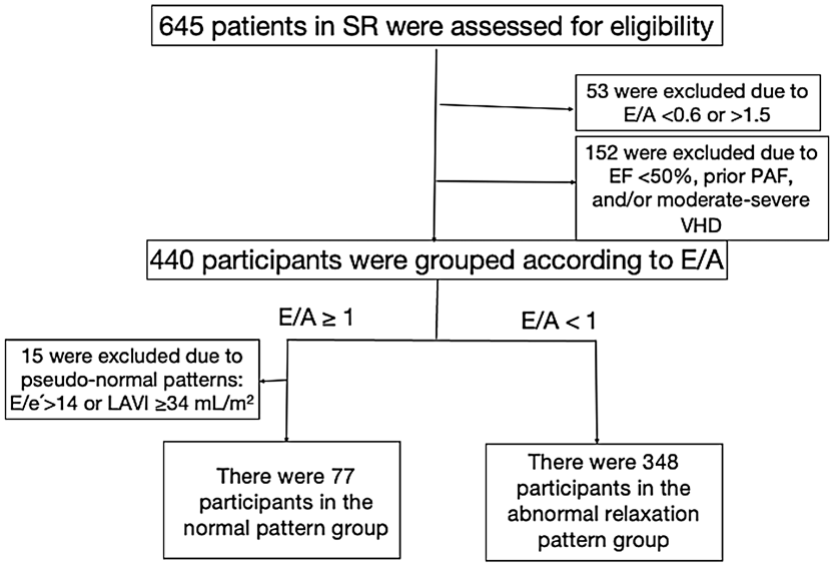
Flow diagram of the patient selection process. LAVI, left atrial volume index; LVEF, left ventricular ejection fraction; PAF, paroxysmal atrial fibrillation; SR, sinus rhythm; VHD, valvular heart disease.

We enrolled 645 consecutive patients with sinus rhythm and without heart failure aged 60–65 years who underwent echocardiography at the Health Co-op Watari Hospital in Japan between January 2006 and December 2009. From this group, we excluded 152 patients with a history of paroxysmal atrial fibrillation, moderate-to-severe valvular heart disease, and/or left ventricular systolic dysfunction (left ventricular ejection fraction [LVEF] < 50%). We also excluded 6 patients with E/A > 1.5, 47 with E/A < 0.6 and 15 with pseudo-normal inflow patterns (E/A > 1, and E/e′ > 14 or LAVI ≥ 34 ml/m^2^). (*e′, peak early mitral annular velocity; LAVI, left atrial volume index.) Finally, we evaluated 425 patients in this study (Fig. 2).

This was a single-center retrospective observational study and complied with the principles of the Declaration of Helsinki. The study protocol was approved by the Ethics Committee of the Health Co-op Watari Hospital (2015–012). Informed consent was obtained by opt-out.

### Echocardiography

Echocardiography was performed in the left lateral decubitus position using the SONOS5500 device (Phillips Medical Systems, Andover, MA, USA). All measurements were performed according to the recommendations of the American Society of Echocardiography^7–9^.

In the parasternal long-axis view, we measured interventricular septal wall thickness (IVS) and posterior wall thickness (PWT) at end diastole. LV end-diastolic diameter (LVDd) and end-systolic diameter (LVDs) were also measured. Left ventricular mass (LVM) and left ventricular mass index (LVMI, g/m^2^) were obtained using the cube formula: LVM (g) = 0.8 × [1.04 (LVDd + PWT + IVS)^3^ − LVDd^3^] + 0.6.

Using the biplane Simpson’s method, the left ventricular volumes at end-diastolic and end-systolic (LVEDV and LVESV, respectively) were measured from the apical 4 and 2 chamber views. LVEF was calculated using the equation:

$${\text{LVEF}}\;{\text{(\% )}} = ({\text{LVEDV}} - {\text{LVESV}}){\text{/LVEDV}}.$$

LAVI (mL/m^2^) was measured using the biplane modified Simpson’s method.

We assessed mitral inflow by pulsed-wave Doppler imaging, i.e., E, A, the deceleration time (Dct) of E, and the E/A ratio were calculated.

Peak early mitral annular velocity (e′) was assessed by tissue Doppler imaging from the apical 4 chamber view. The E/e′ ratio was also determined.

Maximal tricuspid valvular regurgitation velocity was measured using continuous Doppler imaging, from which, the right ventricular pressure (RVp) was estimated using the formula:

$${\text{eRVp}}\;{\text{(mmHg)}} = {4} \times {\text{TRV}}^{{2}} + {1}0.$$

### Classification of mitral inflow patterns

The patients were divided into two groups according to their mitral inflow pattern: the normal pattern group and the abnormal relaxation pattern group. The normal pattern was defined as higher E than A (E/A ≥ 1) with normal LAVI (≤ 34 mL/m^2^) and E/e′ (≤ 14). The abnormal relaxation pattern was defined as lower E than A (E/A < 1). Among the patients with E/A ≥ 1, 15 patients with high left ventricle filling pressure (E/e′ > 14) or a larger left atrium (LAVI ≥ 34 L/m^2^) were excluded for having a pseudo-normal pattern^3,10^ after we performed the Valsalva maneuver^10^.

### Cardiovascular events during follow-up

All patients were followed up on a long-term basis for the incidence of cardiovascular (CV) events. Endpoints were cardiac death, nonfatal myocardial infarction, unstable angina pectoris, heart failure, atrial fibrillation, and fatal arrhythmias. These CV events were assessed using the data from our hospital’s medical records.

### Statistical analysis

Statistical analyses were performed using the JMP® software package for Mac v.13 (SAS Institute, Tokyo, Japan). Values were expressed as means ± standard deviations. Differences between the two groups were evaluated by Student’s *t*-test and chi-squared test for continuous and categorical variables, respectively. The relationships between inflow pattern and the other parameters were evaluated by logistic regression analysis. Event-free survival curves were constructed using the Kaplan–Meier method and compared with log-rank tests. Hazard ratios with 95% confidence intervals (CIs) for CV events attributable to transmitral inflow patterns were calculated using the Cox proportional hazard model. A *p*-value of < 0.05 was considered statistically significant.

## Results

### Baseline characteristics

There were 425 patients in our sample, 77 in the normal pattern group and 348 in the abnormal relaxation pattern group.

Table 1 shows the baseline clinical characteristics of our patients. There were no significant differences in age or gender between the two groups. However, there was a significantly lower average body mass index (BMI) (22.3 ± 3.1 vs. 23.9 ± 3.7, *p* < 0.001), a lower incidence of hypertension (37.7% vs. 57.5%, *p* = 0.002) in the normal pattern group than in the abnormal relaxation pattern group. However, there were no differences in the incidence of dyslipidemia, diabetes mellitus, smoking, chronic kidney disease (CKD), or ischemic heart disease between the two groups.

**Table 1:** Baseline patient characteristics.

	Normal pattern group (n = 77)	Abnormal relaxation pattern group (n = 348)	*p*-value
Age, years	62.4 ± 1.8	62.5 ± 1.8	0.71
Male, n (%)	45 (58.4)	199 (57.2)	0.84
BMI	22.3 ± 3.1	23.9 ± 3.7	< 0.001
HT, n (%)	29 (37.7)	200 (57.5)	0.002
DLp, n (%)	22 (28.6)	117 (33.6)	0.39
DM, n (%)	25 (32.5)	90 (25.9)	0.24
Smoker, n (%)	1 (1.3)	15 (4.3)	0.21
CKD, n (%)	2 (2.6)	30 (8.6)	0.07
IHD, n (%)	2 (2.6)	12 (3.4)	0.71
**Medications, n (%)**			
Calcium blocker	14 (18.2)	117 (33.6)	0.008
ACE-I/ARB	14 (18.2)	115 (33.0)	0.01
Beta-blocker	4 (5.2)	29 (8.3)	0.35
Diuretics	4 (5.2)	21 (6.0)	0.78
Statins	11 (14.3)	60 (17.2)	0.53
Antiplatelets	3 (3.9)	15 (4.3)	0.87

ACE, angiotensin-converting enzyme inhibitor; ARB, angiotensin II receptor blocker; BMI, body mass index; CKD, chronic kidney disease; DLp, dyslipidemia; DM, diabetes mellitus, HT, hypertension; IHD, ischemic heart disease.

The receipt of medical treatment with calcium blockers and angiotensin-converting enzyme inhibitor/angiotensin II receptor blockers was significantly lower in the normal pattern group.

### Baseline echocardiographic findings

Table 2 shows the baseline echocardiographic measurements. There was no difference in the LVDd, LVDs, LVEF, or LAVI between the two groups. However, the normal pattern group had a significantly lower average heart rate (58.5 ± 7.5 vs. 66.2 ± 9.3 bpm, *p* < 0.001), IVS (0.9 ± 0.1 vs. 1.0 ± 0.2 cm, *p* = 0.007), PWT (0.9 ± 0.1 vs. 1.0 ± 0.2 cm, *p* = 0.008), and LVMI (87.4 ± 19.9 vs. 94.3 ± 24.5 g/m^2^, *p* = 0.01) than the abnormal relaxation group. Doppler study measurements found e′ to be higher in the normal pattern group than in the abnormal relaxation group (8.2 ± 1.7 vs. 6.6 ± 1.5 cm/s, *p* < 0.001), but there was no difference in E/e′ or eRVp between groups.

**Table 2:** Baseline echocardiographic results.

	Normal pattern group	Abnormal relaxation pattern group	*p*-value
Heart rate, bpm	58.8 ± 7.5	66.2 ± 9.3	< 0.001
LVDd, cm	4.5 ± 0.5	4.5 ± 0.5	0.41
LVDs, cm	2.9 ± 0.3	2.9 ± 0.3	0.51
IVS, cm	0.9 ± 0.1	1.0 ± 0.2	0.007
PWT, cm	0.9 ± 0.1	1.0 ± 0.1	0.008
LVEF, %	64.9 ± 3.2	64.7 ± 3.7	0.78
LAVI, mL/m^2^	23.0 ± 5.2	23.9 ± 7.2	0.20
E, cm/s	72.1 ± 12.9	58.5 ± 11.7	< 0.001
Dct, ms	195.2 ± 36.0	226.9 ± 50.4	< 0.001
A, cm/s	64.5 ± 11.5	75.0 ± 14.5	< 0.001
E/A	1.12 ± 0.12	0.79 ± 0.11	< 0.001
e′, cm/s	8.2 ± 1.7	6.6 ± 1.5	< 0.001
E/e′	9.1 ± 2.1	9.2 ± 2.7	0.75
LVMI, g/m^2^	87.4 ± 19.9	94.3 ± 24.5	0.01
eRVp, mmHg	22.1 ± 6.8	24.5 ± 7.5	0.13

A, peak late transmitral velocity; E, peak early transmitral velocity; e′, peak early mitral annular velocity; eRVp, estimated right ventricular pressure; Dct, deceleration time; IVS, interventricular septal thickness; LAVI, left atrial volume index; LVDd, left ventricular diastolic diameter; LVDs, left ventricular systolic diameter; LVEF, left ventricular ejection fraction; LVMI, left ventricular mass index; PWT, posterior wall thickness.

### Univariate and multivariate logistic regression analysis of the relationship of patient variables with normal inflow pattern

Univariate analysis found significant relationships between normal inflow patterns and BMI, hypertension, and heart rate. CKD was not significantly correlated with inflow patterns. Multivariable logistic regression analysis found the normal inflow pattern to be associated with odds ratios of 0.859 for BMI (95% CI 0.788–0.937), 0.529 for hypertension (98% CI 0.303–0.924), and 0.228 for heart rate (95% CI 0.228–0.463) (Table 3).

**Table 3:** Univariate and multivariate analysis of the relationship of clinical variables with normal inflow pattern.

	Univariate analysis		Multivariate analysis	
	Odds ratio	95% CI	Odds ratio	95% CI
BMI/SD	0.873	0.809–0.942	0.859	0.788–0.937
HT	0.447	0.269–0.743	0.529	0.303–0.924
CKD	0.283	0.066–1.209		
Heart rate/SD	0.343	0.244–0.482	0.325	0.228–0.463

BMI, body mass index; CI, confidence interval; CKD, chronic kidney disease; HT, hypertension; SD, standard deviation.

### Cardiovascular events during follow-up

All patients were followed up for 1–6 years (4.9 ± 1.8 years). During the follow-up period, 30 CV events occurred (1 CV event in the normal pattern group and 29 in the abnormal relaxation pattern group, comprising 9 cardiac deaths, 3 nonfatal acute myocardial infarctions, 5 unstable angina pectoris, 2 heart failures, 10 atrial fibrillations, and 1 ventricular fibrillation). In particular, only one CV event (one cardiac death) was observed in the normal group (Table 4).

**Table 4:** Cardiovascular events among patients during the follow-up period.

Events	Normal pattern group	Abnormal relaxation pattern group
Total CV events	1	29
Cardiac death	1	8
Nonfatal myocardial infarction	0	3
Unstable angina pectoris	0	5
Heart failure	0	2
Atrial fibrillation	0	10
Fatal arrhythmia	0	1

CV, cardiovascular.

The incidence rates of CV events during the follow‐up period were 0.25%/387 person‐years in the normal pattern group and 1.72%/1685 person-years in the abnormal relaxation pattern group. Using the Cox proportional hazard model, the multivariate (gender, heart rate, BMI, and presence of hypertension)-adjusted hazard ratio for CV events was significantly lower in the normal pattern group (HR: 0.119, 95% CI 0.016–0.910) (Table 5).

**Table 5:** Incidence rates and hazard ratios of cardiovascular events in relation to mitral inflow pattern.

	Normal pattern group	Abnormal relaxation pattern group
Number of patients, n	77	348
Person-year	387	1685
Number of CV events, n	1	29
Incidence rate/1000 person-year	2.5	17.2
Hazard ratio (95%CI)	0.148 (0.020–1.085)	1.0
Gender- and heart rate-adjusted hazard ratio	0.138 (0.018–1.048)	1.0
Multivariate-adjusted* hazard ratio	0.119 (0.016–0.910)	1.0

CI, confidence interval; CV, cardiovascular.

*Adjusted for gender, heart rate, body mass index, and presence of hypertension.

**Figure 3:**
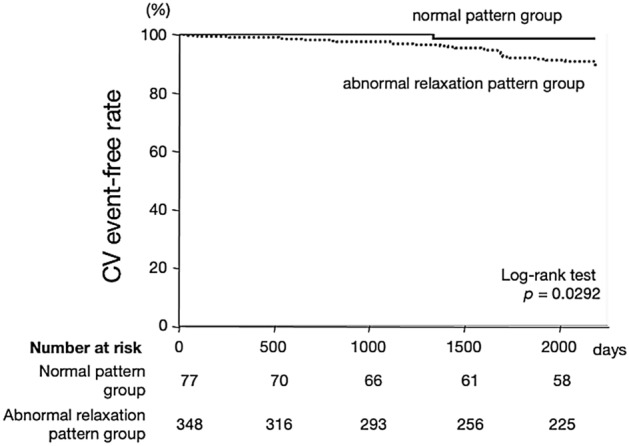
Kaplan–Meier survival analysis of cardiovascular (CV) event-free rates. The CV event-free rates were significantly decreased in the normal inflow group compared to the abnormal relaxation group (*p* = 0.0292).

Figure 3 shows the event-free Kaplan–Meier curves for CV events. A significantly lower incidence of CV events occurred in the normal pattern group than in the abnormal relaxation pattern group (log-rank test = 0.0292).

## Discussion

Our main findings were as follows: (1) Early advanced-age individuals with a normal mitral inflow pattern showed significantly lower BMI, heart rates, and prevalence of hypertension than those with an abnormal relaxation mitral inflow pattern. (2) A normal mitral inflow pattern in early advanced-age individuals without heart failure predicts better prognoses following CV events. To the best of our knowledge, this is the first study to determine that a normal mitral inflow pattern (1.0 < E/A < 1.5) indicates a superior prognosis compared to those with an abnormal relaxation pattern (0.6 < E/A < 1.0) in early advanced-age individuals with normal left ventricular systolic and diastolic functions.

### Characteristics of subjects with a normal mitral inflow pattern

In this study, the characteristics of individuals with normal mitral inflow patterns were lower BMIs, heart rates, and prevalence of hypertension.

Previous research has reported a negative correlation between E/A and BMI^11,12^. Hirokawa et al. showed that age-adjusted E/A is correlated with BMI in those without cardiac disease or atherosclerotic risk factors and found that a rise in BMI increases total blood volume and leads to LV hypertrophy and eventual LV diastolic dysfunction^11^. Other studies have shown that obesity induces systemic inflammation and leads to cardiomyocyte hypertrophy and interstitial fibrosis through inflammation of the coronary microvascular endothelium^13^. Our data suggest that a lower BMI helps to prevent impairment of diastolic function in early advanced-age subjects without heart failure.

Diastolic function is affected by heart rate^14–16^, and heart rate showed the strongest correlation with mitral inflow pattern in our study. A systematic review by Palatini et al. demonstrated that blood pressure, blood glucose, and BMI are positively correlated with HR^17^. Reil et al.^15^ also found that heart rate reduction by I_f_-inhibition improved vascular stiffness, LV contractility, and diastolic function. In patients with decompensated heart failure, a higher heart rate significantly increases Ees and decreases LV-arterial coupling^18^. Inoue found that a higher heart rate leads to hypertension in normotensive individuals^19^, while Shigetoh et al. found that a higher heart rate predicts the development of obesity, diabetes mellitus, and insulin resistance^20^. Studies suggest a relationship between heart rate and activation of the sympathetic nervous system. Higher heart rate leads to sustained pulsatile stress, which causes stiffening of the arterial walls and beta-adrenergic stimulation, leading to various cardiovascular risk factors, accelerating atherosclerosis, and elevating blood pressure. Hypertension and/or other cardiovascular risk factors then lead to diastolic heart failure^21,22^.

Our data showed that a normal mitral inflow pattern in older adults is related to a lower heart rate and reduced prevalence of hypertension. This may indicate low sympathetic nervous system activation in those with a normal inflow pattern.

### Cardiovascular events in the follow-up period in those with a normal mitral inflow pattern

We found a normal mitral inflow pattern in early advanced-age subjects without heart failure to predict superior prognoses following CV events and reduced incidence of such events (1 < E/A < 1.5) than in those with an abnormal relaxation pattern (0.6 < E/A < 1).

Several investigators have demonstrated the prognostic value of the mitral inflow pattern in population-based cohorts. Bella et al. compared the prognosis of three groups of middle-aged and elderly adults, those with E/A of < 0.6, 0.6–1.5, and < 1.5^23^. They found that an E/A > 1.5 is associated with increased mortality. Fox et al. found E/A < 0.7 or > 1.5 to be associated with increased mortality compared to a control group (E/A: 0.7–1.5)^24^. However, none of these studies demonstrated whether a subgroup of early advanced-age individuals with E/A between 0.6 and 1.5 has a superior prognosis for CV events.

In our study, we focused on individuals with E/A between 0.6 and 1.5 and excluded those with diastolic dysfunction, manifested in an abnormal relaxation pattern with an E/A < 0.6, a restrictive pattern (E/A > 1.5), or a pseudo-normal pattern. Our results showed that the subgroup of “supernormal inflow pattern,” i.e., 1.0 < E/A < 1.5, had a superior prognosis in those of early advanced age.

Resting E/A is strongly correlated with exercise tolerance in younger populations and elderly healthy athletes^25^. There are some reports that lifelong regular exercise may reduce the effects of aging on the heart^25–28^. Seniors with a lifelong history of vigorous exercise training maintain youthful LV compliance and distensibility compared to healthy sedentary seniors^29^. Superior prognoses in early elderly patients with normal inflow patterns may be related to exercise tolerance.

### Is a normal inflow pattern a “young heart” marker in early advanced-age subjects?

Changes to the heart due to aging include an increase in the LVMI and a decrease in the LV diastolic function^5^. In our study, the participants with a normal inflow pattern also had lower BMI and heart rates and no hypertension and showed superior clinical outcomes. Therefore, a normal inflow pattern may be the marker of a “young and supple heart” in early advanced-age subjects with normal left ventricular systolic and diastolic functions.

### Study limitations

This study had some limitations. First, this is a retrospective single-center study involving a small sample population. Therefore, our findings will need to be confirmed in studies with a larger sample size and multi-center verification. Second, regular exercise habits and exercise tolerance were not evaluated at baseline. Thus, it remains unclear whether exercise improves prognosis in early advanced-age individuals. Third, we did not investigate whether the E/A changes after treatment for hypertension or a decrease in BMI. Thus, it is unclear whether a patient’s prognosis changes in line with changes in E/A. Finally, we did not investigate the effects of sympathetic nervous system activation. The above omissions all require investigation in future research.

## Conclusion

A normal inflow pattern in early advanced-age individuals is associated with a lower BMI, an absence of hypertension, and a lower heart rate and predicts a better prognosis following cardiovascular events.

## Data Availability

The datasets used and/or analysed during the current study available from the corresponding author on reasonable request.
